# The potential role of short chain fatty acids improving *ex vivo* T and CAR-T cell fitness and expansion for cancer immunotherapies

**DOI:** 10.3389/fimmu.2023.1083303

**Published:** 2023-01-20

**Authors:** Adrián González-Brito, Mireia Uribe-Herranz

**Affiliations:** Institut d’Investigacions Biomèdiques August Pi i Sunyer (IDIBAPS), Hospital Clinic de Barcelona, Barcelona, Spain

**Keywords:** immunotherapy, short chain fatty acids, car-t, T cells, *ex vivo*, gut microbiota

## Abstract

Adoptive cell therapies, like tumor-infiltrating lymphocytes or chimeric antigen receptor T cells, have become an important immunotherapeutic approach against cancer. One of the main struggles of T cell immunotherapies is how to obtain the most effective T cell phenotype, persistence, and differentiation potential to infuse into patients. Adjusting the T cell *ex vivo* cell culture conditions is a key factor to increase and improve the efficacy of cellular immunotherapies. In this review, we have summarized the *ex vivo* impact of short chain fatty acids, a group of gut microbiota derived metabolites, on T cell culture and expansion for immunotherapies. There is a complex gut microbiota-immune system interaction that can affect antitumor immunotherapy efficacy. Indeed, gut microbiota derived metabolites can modulate different biological functions in the immune system local and systemically.

## Introduction

According to the World Health Organization, cancer is the second leading cause of death worldwide with almost 10 million deaths in 2020. Cancer immunotherapy has rapidly become one of the cornerstones of cancer therapy along with surgery, radiation therapy and chemotherapy. It works by stimulating the immune system to recognize and kill cancer cells and control tumour growth in a way that prevents damage to the healthy cells. It is agreed that modern cancer immunotherapy began with the publication in 1893 of the first use of Coley’s bacterial toxins to treat tumours (1). Since then, many different types of immunotherapies against cancer have been developed, from stem cell transplants, monoclonal antibodies, and immune checkpoint inhibitors to cellular immunotherapies like cancer vaccines or T-cell transfer therapy. Adoptive cell therapies represent a promising and fast evolving approach for the treatment of cancer and include tumour-infiltrating lymphocytes (TILs) (2) or chimeric antigen receptor (CAR) T cells (3). In the last decade, the CAR T-cell therapy has yielded exceptional efficacy rates for the treatment of several hematologic malignancies (4–8). CAR-T cells are genetically modified T cells that combine, an antigen recognition domain of a specific antibody with an intracellular domain of the CD3-z chain protein into a single chimeric protein region that promotes T-cell cytotoxic activity and proliferation. Hence, CAR-T cells can target the very same antigen expressed in the tumoral cell surface, in a major histocompatibility complex (MHC)-independent manner. There have been ground-breaking responses to treatment but, so far, CAR-T cell therapy is available and effective only for a minority of patients. The main concerns regarding treatment efficacy are CAR-T cell differentiation potential, proliferation, and persistence (9). There are multiple approaches attempting to address these problems from the manipulation of CAR constructs, the selection of T-cell subset populations, pharmacological inhibitors, or the optimization of the CAR T-cell manufacturing process. This last approach will be the focus of this review.

Tuning the *ex vivo* cell culture conditions is key to determine differentiation status and survival of CAR-T cells. Developing the desired CAR-T profile, undifferentiated, less exhausted, and persistent, is an area of intense research. One of the first options to improve the T-cell fitness is the choice of the T-cell source to manufacture the CAR-T cells. It has been extensively shown that T-cell origin has a critical impact on the CAR-T biological activity and proliferation [reviewed in (10)]. Besides the source of T cells and T-cell subtypes, multiple additional strategies are being tested to tune the *ex vivo* cell culture conditions; the manufacturing time, the stimulation with cytokines, the T-cell activation, the gene delivery system, and strategies to target or reprogramme the T-cell metabolism.

Currently, protocols to manufacture CAR-T cells are varied and long, taking up to several weeks. In our experience, with a locally developed academic anti-CD19 CAR-T (ARI-0001) for a clinical trial, the expansion takes between 9 and 12 days (11). So far, no standardization has been reached in the field. A key aspect that differs between protocols is the stimulation with cytokines, being the most common and studied interleukin 2 (IL-2), 7 (IL-7), 15 (IL-15) and 21 (IL-21). IL-2 stimulates cell proliferation and maintains viability during the *ex vivo* expansion (12). However, since stimulation with IL-2 can also induce an exhausted T-cell profile, a well-known alternative is to switch it for IL-7, IL-15, and IL-21, with the aim of promoting naïve and memory cell proliferation and preventing T-cell differentiation (13). Regarding T-cell activation strategies, the most employed are the anti-CD3/anti-CD28 monoclonal antibodies either soluble, bound or in magnetically coated beads, which act as artificial antigen presenting cells. The gene transfer system is another component that can be modified during the CAR-T cell *ex vivo* manufacturing. Currently, the usual choice is a viral vector because of its high transduction efficacy. The most common system used in the available manufactured CAR-T therapies are the lentivirus, followed by retrovirus. However, viral gene transfer has a major disadvantage. Besides the limitation in gene size (<10 Kb), the insertion of the gene occurs randomly which could potentially lead to oncogenesis through the activation of an oncogene or suppression of a tumor-suppressor gene. To avoid this issue, non-viral systems are being developed such as plasmid-based gene delivery using the transposon/transposase systems (14, 15) or CRISPR/Cas9-based gene editing (16).

Another important area of research is optimizing metabolism at different stages of CAR−T cell production. A low metabolic activity during the manufacturing process correlates with a less differentiated T cell phenotype, longer persistence *in vivo* and greater antitumoral activity (17). On the other hand, a high metabolic activity has been linked to a more differentiated profile (18). To optimize metabolism, several approaches are being tested during T cell expansion, such as inhibiting glycolysis to limit differentiation and improve T cell function or enhancing mitochondrial metabolism (17, 19–21). Finally, a new area of improvement has been developing for the last 5 years, as few studies are starting to use metabolites of bacterial origin, commonly found in the human body, to modulate T-cell phenotypes *ex vivo* (**Table 1**).

**Table 1:** *ex vivo* acetate, propionate, butyrate and pentanoate impact on diverse T cell subsets.

		Acetate		Propionate		Butyrate		Pentanoate
	T cell	Low	High	Low	High	Low	High	High
CD4	Tregs	= FoxP3 (22)		↑FoxP3 (22, 23)	= FoxP3 (23)	↑↑ FoxP3 (22, 24)	= FoxP3 (24)	
	Th1	↑↑Th1 profile ↑IL10 secretion (25)		↑↑Th1 profile ↑IL10 secretion (25)			↑↑Th1 profile ↑IL10 secretion (25)	
	Th17	↑↑Th17 profile ↑IL10 secretion (25)	↑ IL10 secretion (26)	↑↑Th17 profile (25) ↑ IL10 secretion (25, 26)			↑↑Th17 profile ↑IL10 secretion (25)	block Th17 polarization (26) ↑ IL10 secretion (26)
	Naive	↑Th17 polarization (25)		↑Th17 polarization (25)				
CD8		= IFNγ (27) = TNFα (27)	↑↑ IFNγ (28)		↑↑IFNγ (28) ↑↑TNFα (27) ↑↑TNFα/IFNγ + CD8 (27)		↑↑IFNγ (28, 29) ↑FoxO1 (30) ↑ Tumor Control (27) ↑↑TNFα/IFNγ (27) ↑ memory phenotype (27)	
**TILs**			↑↑ IFNγ (31)					
**CARTs**							↑↑IFNγ (27) ↑TNFα (27) ↑CD25 (27)	↑↑IFNγ (27) ↑↑TNFα (27) ↑CD25 (27) ↑IL2 (27) ↑Tumor control (27) ↑Cytolytic activity (27) ↑Proliferation (27)

Acetate, low < 5mM and high ≥ 5mM concentration. Propionate, low < 0.4 mM, and high ≥ 0.8 mM concentration. Butyrate, low < 0.25 mM, and high ≥ 0.5 mM concentration. Pentanoate, high ≥ 0.5 mM concentration.

The human body is a complex ecosystem colonized by trillions of bacteria, fungi, yeast, protozoa, and viruses. All these together comprise the commensal microbiota that is developed after birth through vertical transmission and then shaped by environmental factors throughout life. The commensal microbiota and the human host have co-evolved in a mutualistic association, obtaining benefits such as the acquisition of bioactive compounds (32). These metabolites can modulate various biological functions in the immune and nervous systems (33). Beyond the effects on intestinal and local immune physiology, the gut microbiome has systemic effects (34) caused by small molecules such as bacterial-derived metabolites entering the systemic circulation. Several studies have confirmed that gastrointestinal flora impacts the immune system predominantly through bacteria-derived metabolites (35, 36). The gut microbiota has great metabolic capacity, greater than that of the human host. It generates a complex network of metabolic pathways that produce an exceptionally diverse pool of metabolites from modified exogenous dietary components to endogenous compounds generated by the gut microbiota itself (35, 37, 38). For example, *in vivo* short chain fatty acid (SCFA) can regulate host immunity by facilitating the extrathymic generation of regulatory T (Treg) cells (39), and the function of the colonic Tregs (40); peptidoglycan, an essential molecule of the bacterial wall, can prime the systemic innate immunity by activating neutrophils (41); polysaccharide (PSA) from *B. fragilis, a* Gram-negative anaerobe, can boost the systemic T helper cell type 1 (Th1) CD4^+^ T cells (42).

The results from an *in vivo* study with mice, raised under germ-free conditions, confirmed that the impact of gut microbiota is not restricted to the gastrointestinal tract. It has systemic effects, as these mice had significantly impaired host immune responses to pathogens (43). Moreover, a direct correlation exists between the presence of specific bacteria in the gut microbiota and T-cell development and differentiation (44–47). For instance, colonization of germ-free mice gut with a cocktail of bacteria from Clostridiales clusters IV, XIVa, and XVIII is sufficient to drive Treg differentiation (45). In the cancer context, some specific bacteria have been demonstrated to be involved in the process of initiation and progression of carcinogenesis at epithelial barriers and within sterile tissue (48, 49). In addition, the microbiota has also been implicated in modulating the efficacy and toxicity of cancer therapy, including chemotherapy, radiotherapy, and especially immunotherapy (32, 50–52), including checkpoint blockade approaches targeting the CTLA-4 and PD-1 pathways (53, 54) mainly acting through the local and systemic immune system. There are several studies that support the link between gut microbiota (or bacterial-derived metabolites), Th differentiation and T cells function, and modulation of the antitumor immunity. A high-dietary fiber diet, which is associated with increased gut microbiota diversity and decreased risk of chronic inflammatory diseases, can boost antitumor immunity and increase the infiltration of tumor-killing T cells on melanoma patients (55). In this observational study, fiber-fermenting *Ruminococcaceae* correlated with the abundance of inducible T cell co-stimulator-expressing TILs on melanoma. In a study investigating four different murine cancer models, inosine, a bacterial purine metabolite produced by *Bifidobacterium pseudolongus*, promoted Th1 activation and antitumor immunity which improved the antitumor effects (56). In a mouse model of colon carcinoma, a SCFA-rich diet with pectin, a fiber that promotes the growth butyrate-producing bacteria, was associated with increased CD8+ effector T cell function at the tumor site (57). A recent study, showed that the natural polyphenol castalagin improved the intratumoral CD8+/FoxP3+CD4+ ratio in sarcoma tumor-bearing mice, and improved the efficacy of anti-PD-1 immunotherapy through modulation of the gut microbiota, enriching the abundance of *Ruminococcaceae* and *Alistipes* bacterial families (58). Another report investigating the immune-checkpoint blockade therapy efficacy, showed that *Lactobacillus delbrueckii subsp. bulgaricus* produced an extracellular polysaccharide that was able to induce IFN-γ+CCR6+CD8+T cells in Peyer’s patches of tumor-bearing mice, thus enhancing the efficacy of this therapy (59). A study from Wang and colleagues, proved that plasma trimethylamine N-oxide, a microbe-derived metabolite, can activate the endoplasmic stress kinase PERK, boosting the function of IFN-γ+CD8+T cells mediated immunity in triple-negative breast cancer patients (60). Besides gut microbiota, intratumoral bacteria also can have a role in antitumor immunity through T cells. *Fusobacterium nucleatum* can attenuate T cell-mediated immune responses in rectal and colon cancer cases, as higher amount of *F. nucleatum* in colorectal carcinoma tissue was associated with lower density of T-cells in tumor tissue (61). All these studies provide strong evidence of a highly dynamic and complex microbiome-immune system interaction that can impact antitumor immunity.

### Short Chain Fatty Acids

SCFAs are the main metabolites produced by gut microbiota through bacterial anaerobic fermentation of non-digestible carbohydrates, such as dietary fiber (62). They are fatty acids with fewer than six carbon atoms, the most abundant of which are acetate (two carbons) and propionate (three), which are mainly produced by members of the *Bacteroidetes* phylum, as well as butyrate (four), predominantly produced by members of the *Firmicutes* phylum. Acetate derived from colonic bacterial fermentation can flow into the blood compartment and together with endogenous acetate can exert systemic activity upon the immune system. Propionate is a precursor of glucogenesis and has a direct effect on T cell activity locally. Butyrate effects are mainly restricted to the gut, where it is used as energy sourced by the colonocytes and, together with propionate, is processed in the liver (63). The effect of SCFAs on the metabolism of T-cells is yet to be understood but there is abundant data that suggest that SCFAs regulate the adaptive immune system through the modulation of mTOR activity, glucose metabolism, histone acetylation and cytokine gene expression (64). Multiple studies have confirmed that T cells are susceptible to SCFA exposure *in vitro*, promoting or inhibiting a specific T cell phenotype, and therefore supporting their use in *ex vivo* T cells expansion.

While research on the effect of microbial metabolites on T cells has primarily been focused on SCFAs, there are also other metabolites that have been investigated. For instance, the inosine, a purine metabolite that can be produced by *Bifidobacterium pseudolongum*, has been shown to boost the CD4+ Th1 differentiation in the presence of IFN-γ through adenosine A2A receptor (56). Moreover, the bacterial transformation of host bile acids has been demonstrated to directly modulate balance of Th17 and Treg cells (65, 66). Furthermore, tryptophan metabolites are reported to be essential for intestinal immunity and perform their effect on T cells through aryl hydrocarbon receptor signaling (67). Although these bacterial-derived metabolites are promising alternatives to SCFAs, in this manuscript, we will focus on the new and fast developing research field of the T and CAR-T cell *ex vivo* manufacturing optimization process through the SCFA bacterial derived metabolites. Results summarized in this review, might appear difficult to interpret as the effects of SCFAs are clearly dependent on immunological context and the studies compared often used a wide range of different conditions, from concentrations to exposure time. This is expected, as the field is only just starting to investigate the effect of gut microbial-derived metabolites on immune populations *ex vivo*. This lack of clarity and consistency reflects the urgent need for more rigorous and systematic protocols to better understand and learn from the microbiome-T cell crosstalk.

## The *ex vivo* effects of SCFAs on T-cells

### Naïve CD4+ T cells

The effect of SCFAs in regulating T cell differentiation into effector and IL-10+ regulatory T cells was studied by Park et al. In this study T cells were activated *in vitro* for 5-6 days in the presence or absence of the SCFAs at different concentrations. The results showed that naïve CD4+ T cells, isolated from murine spleens and lymph nodes (LN), treated with acetate and propionate *in vitro*, led to differentiation into Th17 cells, which was observed through the increase in IL-17+ and IL-10+ cells as well as the increase in the transcription of the associated genes *IL-17A, IL-17F, Rorc, RORα, T-bet, and IFN-g* (25) **Tables 1**, **2**.

**Table 2:** Summary of methods and results from *ex vivo* SCFA effects on murine T cell studies.

Article	T cell subset	Source	Activation	Polarization	Metabolites	Timing and duration of treatment	Effect	Mechanism
Furusawa et al. (22)	CD4+ T cells	Sp and LN C57BL/6 mice	p-c anti-CD3 (10 μg/ml) and soluble anti-CD28 (1 μg/ml)	Tregs: TGF-β1 (0.2 ng/ml), IL-2 (10 ng/ml) Th1 cells: IL-12 (10 ng/ml), anti-IL-4 (10 μg/ml) Th2 cells: IL-4 (10 ng/ml), anti-IL12 (10 μg/ml) Th17: TGF-β1 (0.2 ng/ml), IL-6 (40 ng/ml), anti-IFN-γ (10 μg/ml) and anti-IL-4 (10 μg/ml)	Acetate (0.1 mM) Propionate (0.1 mM) Butyrate (0.1 mM)	1-3 days, some experiments during polarization, in some experiments after 2- or 3-day culture	Butyrate significantly increased the concentration of Foxp3+ cells, even under Th1- and Th17-polarizing conditions	histone H3 acetylation of the Foxp3 locus
Park et al. (25)	CD4+ T cells CD8+ T cells	Sp and LN C57BL/6 mice	p-c anti-CD3 (5 μg/ml) and soluble anti-CD28 (2 μg/ml)	Th17/Tc17: rhTGF-β1 (5 ng/ml), mIL-6 (20 ng/mL), mIL-1b (10 ng/ml), mIL-23 (10 ng/ml) mIL-21(10 ng/ml), mTNF-a (20 ng/ml), anti-mIL-4 (10μg/ml), and anti-mIFN-γ (10μg/mL) Th1/Tc1 cells: hIL-2 (100 U/ml), mIL-12 (10 ng/ml), and anti-mIL-4 (10μg/ml) Non-polarized: hIL-2 (100 U/ml)	Acetate (1-20 mM) Propionate (0.1-1 mM) Butyrate (0.125-0.5 mM)	During differentiation for 5-6 days	Enhanced production of IFN-γ and IL-17 in Th1/Tc1 and Th17/Tc17 polarising conditions. SCFAs promoted differentiation of CD4+ and CD8+ T cells, into IL-10 producers in all polarization conditions	HDAC inhibition and regulation of the mTOR–S6K pathway (acetate and propionate)
Kespohl et al. (24)	CD4+ T cells	Sp and LN C57BL/6 mice	p-c anti-CD3 (5 μg/ml), soluble anti-CD28 (1 μg/ml) 50 U/ml rh IL-2	Th1 cells: rmIL-12 (10 ng/ml), anti-IL-4 Th2 cells: rmIL-4 (40 ng/ml), anti-IFN-γ (10 μg/ml) Th17: rhTGF-β1 (0.5 ng/ml), IL-6 (20 ng/ml), anti-IFN-γ (10 μg/ml), and anti-IL-4	Acetate (0.1-1 mM) Propionate (0.1-1 mM) Butyrate (0.1-1 mM)	2-3 days treatment after 3 days of activation	Low Butyrate concentration at enhances the expression of FoxP3 under suboptimal TGF-B1 conditions. Butyrate and Propionate upregulates IFN-γ in CD4+ T cells at 1 mM.	Hyperacetylation of pro-inflammatory genes
			p-c anti-CD3 (5 μg/ml), soluble anti-CD28 (0.5 μg/ml) 100 U/ml rhIL-2	Tregs: rhTGF-B1 (0.5, 1 or 2 ng/ml), anti-IFN-γ (10 μg/ml) and anti-IL-4 (anti-IL4 at 10% culture supernatant)				
Luu et al. (29)	CD8+ T cells	Sp and LN C57BL/6 mice	p-c anti-CD3 (5 μg/ml), soluble anti-CD28 (1 μg/ml)	CTLs: rhIL-2 (50 U/ml), anti-IFN-γ (10 μg/ml) Tc17 cells: rhTGF-β1 (1 ng/ml), IL-6 (40 ng/ml), anti-IFN-γ (5 μg/ml) Tregs: rhTGF-B1 (2 ng/ml), rhIL-2 (100 U/ml) and anti-IFN-γ (5 μg/ml)	Acetate (1-25 mM) Propionate (0.5-2.5 mM) Butyrate (0.25-1 mM)	3 days treatment after 3 days of activation	1 mM Butyrate and 1 mM Propionate upregulate IFN-γ and Granzyme B for CTLs and Tc17s 1mM Butyrate reduced IL-17 production in Tc17s and switched Tc17s to CTL phenotype Effect of butyrate on CTLs was maintained 5 days after treatment removal	HDAC inhibition (Butyrate) AKT/mTOR pathway (Acetate)

Sp, spleen; LN, Lymph node; p-c, plate-coated; IL, interleukin; CTLs, cytotoxic T Lymphocytes.

### Polarized CD4+ T cells

Studies over the last 10 years have shown the ability of SCFAs to modulate and alter polarized CD4+ T cells *via* epigenetic and metabolic processes. Under tolerogenic conditions, naïve CD4+ T cells supplemented with low or physiogical concentrations of SCFAs seem to play a role in facilitating the activity of T regulatory cells, promoting the production of IL-10 and FoxP3+ Treg polarization (22, 39). Furusawa et al. isolated naïve CD4+ T cells from murine spleen and LNs. After three days of expansion cells were differentiated into Tregs in the presence or absence of acetate, propionate or butyrate, for an additional 2 days. Butyrate significantly increased the concentration of Foxp3+ cells; propionate did so moderately, while acetate showed no effect. The butyrate effect was in part mediated by histone H3 acetylation of the Foxp3 locus (22). Another study provided additional evidence indicating an increase of Foxp3+ CD4+ T cells for lower concentrations of propionate but not for higher ones (23). A third independent study, partially confirmed the enhanced Foxp3 expression in purified murine CD4+ T cells using a suboptimal concentrations of TGF-β1 and low concentrations of butyrate. However, the effect was not observed in the absence or under optimal concentrations of TGF-β1. In any case, higher concetrations of Butyrate did not enhance the expression of Foxp3 and instead, induced the expression of T-bet and IFN-γ *via* histone acetylation (HDAC inhibition), which is associated with an inhibition of Treg differentiation (24).

Luu and colleagues polarized CD4+ T cells towards Th17 for three days. Supplementation with pentanoate, another SCFA with 5 carbons, during the three-day process, impeded the polarization and inhibited the production of IL-17A through HDAC-inhibitory activity. In this case, and contrary to the results for the shorter SCFAs used in the study from Park et al., pentanoate led to a reduction in the transcription of the Th17 associated genes *IL-17A, Rorc, Il21, Stat3, and Tgfb3*. The treatment did, however, lead to the increase in the expression of IL-10 in Th17 cells. This increase seems to be due to pentanoate acting as a precursor of acetyl-CoA and activating the mTOR pathway, similarly to the mechanism for acetate. Results showed that Th17 cells treated *ex vivo* with pentanoate increased the extracellular acidification rate and enhanced their glycolytic activity leading to metabolic and epigenetic reprogramming and the loss of the pathogenic phenotype of Th17 cells in autoimmune disease (26).

Finally, under Th1 CD4+ polarizing conditions in the presence of IL-12, acetate and propionate supplementation potentiated the differentiation into Th1 cells, in a concentration dependent manner (25) **Table 1**.

### Activated CD8+ T cells

Activated CD8+ T cells cultivated *in vitro* using a SCFA-enriched medium have an increased production of IFN-γ and Granzyme B. SCFAs with shorter chain lengths such as acetate (two carbon chain) require greater concentrations for similar effects to ones with longer chain lengths such as pentanoate (five carbon chain) (28–31). For instance, murine CD8+ T cells polarized to CTLs in culture for three days, were supplemented with acetate for an additional three days at a concentration 25 mM. This led to a 125% increase in IFN-γ cells in comparison to untreated cells. Propionate, which contains a three-carbon chain, seems to require a concentration of approximately 5 mM to achieve similar effects (28). To obtain a comparable effect using butyrate, a SCFA with a four-carbon chain, a concentration of only 1 mM was required (29). In line with this hypothesis, murine CD8+ T expansion under CTL-inducing conditions supplemented with low concentration of acetate showed no effect over TNF-α or IFN-γ expression (27). SCFAs of shorter chain lengths are found in the body at greater concentrations (68). This might explain why T cells may could have evolved to become less sensitive to SCFAs of shorter chain lengths through a variety of mechanisms. In the example above, while acetate caused the increase in IFN-γ *via* the mTOR pathway (rapamycin, an mTOR inhibitor, led to an abrogation of the effect), butyrate caused it *via* the inhibition of HDAC activity (29). CD8+ T expansion under CTL-inducing conditions and supplemented with pentanoate, butyrate and, to a lesser extent, propionate showed an increase in the frequencies of TNF-α+ IFN-γ+ CD8+ T cells and secretion of TNF-α by CTLs. The effectiveness of butyrate and pentanoate treated T cells was further tested in a B16-OVA melanoma murine model and showed a better melanoma tumor control. Moreover, pentanoate-treated CD8+ T cells, showed an increase in *in vivo* persistence in a rag1-deficient mouse model, which was associated to an increase in CD25 expression and continuous IL-2 secretion (27). Qiu et al. tested CD8+ T cells under prolonged glucose restriction *in vitro* and showed that supplementation of acetate increased the expression of IFN-γ, although Granzyme B levels were not altered. These effects were mediated through histone acylation (31).

Differences in SCFAs concentration in medium lead to differences the expression of cytokines, however, the effect on the length of exposure seems to be less clear. While Luu and colleagues exposed murine activated CD8+ T cells to 1 mM Butyrate for three days (29), He and collaborators exposed activated CD8+ T cells to butyrate at the same concentration for just 12 hours, with the inclusion of IL-12 (28). They both obtained similar results for the proportion of IFN-γ cells, indicating a doubling in relation to untreated cells.

There are other factors to consider beyond concentrations or the duration of treatment, like whether the effect is long-lasting and leads to stable phenotypic change or their memory potential. The first one was explored by treating CTLs with butyrate for two days and then allowing the cells to grow without it for an additional three days. An increase in IFN-γ was still observed after treatment withdrawal compared to the control group, *in vitro* and *in vivo* , indicating a phenotypic stable change (29). The relationship between SCFA T cell *ex vivo* treatment and their memory potential were studied with transgenic gBT-I CD8+T cells treated with butyrate for three days after antigen-specific activation. While butyrate treatment reduced proliferation, it also led to greater responsiveness to IL-15 and a higher expression of transcription factor FoxO1 (30). Expression of FoxO1 is involved in the formation of memory T cells (69–71) while IL-15 promotes memory CD8+ T-cell survival and proliferation (72, 73), suggesting that butyrate may lead to a greater memory potential of the activated CD8+ T cells. This was supported by *in vivo* experiments that showed that butyrate treated gBT-I CD8+ T cells had a greater expansion upon antigen encounter. The suggested mechanism behind butyrate enhancement of the memory potential of CD8+ T cells might be its impact over glycolytic metabolism, uncoupling the TCA cycle from glycolitic input and favouring oxidative phosphorylation (30) **Table 1**.

### TILs and CAR-T cells

All the studies mentioned above prove that SCFAs can modulate T cell polarization and memory formation *ex vivo*. There are few reports that show better therapy outcomes polarizing CAR-T cells *in vitro* before infusion. Interestingly, Th17-polarized human mesothelin-CAR T cells exhibited superior immunity against mesothelioma compared to Th1-polarized mesothelin -CAR T cells (74). In the same line of results, Guedan and colleagues showed that mesothelin-specific CAR Th17/Tc17 cells (that maintained Th17 function with a Th1 bias) had long-lived persistence *in vivo* and eradicated tumors (75). A third report studying the Th17 polarization effects on CAR-T cells, showed that a Th/Tc17 CAR-T targeting the proto-oncogene Neu, exhibited an increase antitumor immunity and improved early tumor control. The combination of the Th/Tc17 Neu-CAR-T and a STING agonist increased the trafficking, persistence, and tumor control in a murine model of breast cancer (76). Supporting the use of Th17 polarizing conditions for CAR-T cells, Fraietta and colleagues showed that CAR-T cells from chronic lymphocytic leukemia complete responders to CD19-CAR T therapy, had an enhanced transcriptomic profile of STAT3/IL-6 signaling, producing a type-17 signature compared with non-responders (77). Finally, a human mesothelin-CAR polarized towards Th9 cells was able to eliminate advanced human ovarian cancer patient-derived xenograft in humanized NSG mice. Regular expanded CAR-T or high doses of Th1+Tc1 polarized CAR-T cells could not achieve the same results (78). The differentiation status of CART cells plays a crucial role for therapeutic success as well. It is now well stablished that a less differentiated profile, like naïve or central memory T cells which have the capacity to persist and proliferate long-term *in vivo* lead to a better clinical outcome (77, 79). All the studies mentioned above highlight the potential of SCFAs over the process of expanding tumor-specific lymphocytes and CAR-T cell manufacturing for cancer immunotherapies. In this line, two studies recently published have explored the use of SCFAs on TILs and CAR-T cells.

Murine tumor-infiltrating lymphocytes (TILs) obtained from B16 melanoma and treated *ex vivo* with acetate showed a significant increase in IFN-γ secretion. This indicates that acetate can promote responsiveness in T cells isolated directly from the tumor microenvironment (31). The effect of butyrate and pentanoate supplementation during CAR-T cell *ex vivo* expansion was investigated under CTL-inducing conditions. Murine and human CD8+ T cells were used to generate a CAR that recognize receptor tyrosine kinase-like orphan receptor 1 (ROR1). Murine CAR-T cells treated with butyrate and pentanoate enhanced expression of CD25, as well as TNF-α and IFN-γ production. Pentanoate treated cells were further tested in a pancreatic tumor model and showed that tumor volume and weight were significantly reduced in comparison to non-treated cells. Later, the authors developed CAR-T cells from healthy human donor CD8+ T cells using a ROR1-specific CAR. After a two-step 17-day stimulation process, CAR-T cells were treated for four days with pentanoate at different concentrations. CAR-T cells pretreated with pentanoate showed upregulation of CD25 expression, IL-2 secretion, and stronger proliferation, as well as greater cytolytic activity when encountering their target antigen ROR1, together with an increase in IFN-γ and TNF-α production (27). **Table 1**.

## Conclusion

During the last decade multiple studies have confirmed the remarkable effect of gut microbiota and its bacterial derived metabolites upon the immune system. Bacterial T cell polarization and function modulation are two of the most studied aspects of the gut microbiota-immune system axis. These are the basis of several strategies to improve efficacy of immunotherapies or to harness its side effects in clinical trials. According to NIH clinicaltrial.gov site there are 50 clinical trials on cancer immunotherapies that include study or intervention regarding gut microbiota. Nevertheless, the clinical implementation has proven to be difficult. Multiple variables are still largely unknown, like the optimal administration route or how to monitor the evolution once its implemented. Moreover, dosing and administration schedule will be key as it is envisioned as a very dynamic therapeutic process. The studies presented in this review show that microbial metabolites can alter T cell differentiation *in vitro*, potentially leading to a phenotype that can increase persistence, cytotoxic activity and, ultimately, anti-tumor effect, **Tables 2**, **3**. While some results might appear contradictory, expansion and polarization T cell *ex vivo* protocols used up to now are inconsistent. Multiple factors are not well defined, for instance the cytokines used in the media culture, the SCFAs concentrations or the duration of the treatment to name just a few examples. Dosing SCFAs might be the most crucial variable to account for, as current available data shows pleiotropic effects of SCFAs upon T cells. Learning from microbiome-T cell crosstalk can help us to develop a more efficient cancer immunotherapy. We foresee the *ex vivo* use of bacterial-derived metabolites as a new method to improve T cell expansion and polarization, directed to cellular immunotherapies with a special interest on TILs and CAR-T cell production.

**Table 3:** Summary of methods and results from *ex vivo* SCFA effects on murine and human T cell studies.

Article	T cell subset	Source	Activation	Polarization	Metabolites	Timing and duration of treatment	Effect	Mechanism
Bachem et al. (30)	CD8+ T cells	Sp gBT-I or OT-I mice	p-c anti-CD3(5 μg/mL) and anti-CD28 (5 μg/mL)	12.5 U/mM IL-2 added from day 2.	Acetate (0.5 mM) Propionate (0.5 mM) Butyrate (0.5 mM and 0.1 mM)	3 days treatment after 3 days of activation	Butyrate 0.5 mM increased the expression of FoxO1 and responsiveness of cells to IL-15	Uncoupling the TCA cycle from glycolysis and increased glutaminolysis
Qiu et al. (31)	CD8+ T cells	Sp and LN OT-I mice (OVA specific)	OVA (100 nm/ml), rhIL-2 (100 U/ml)	25 mM glucose for 3 days, then switched or not to 1 mM glucose	Acetate (5 mM)	1 or 5 days (during glucose restriction) after 3 days of activation	Acetate rescues effector function in glucose restricted cells and increases IFN-γ production by exhausted CD8+ T cells	Histone acetylation acetyl-CoA synthetase
Luu et al. 2019 (64)	CD4+ T cells	Sp and LN C57BL/6	Th17s: p-c anti-CD3 (5 μg/ml), soluble anti-CD28 (1 μg/ml)	Th17: TGF-β1 (1 ng/ml), IL-6 (25 ng/ml), anti-IFN-γ (5 μg/ml), and anti-IL-4 Tregs: TGF-β1 (2 ng/ml), anti-IFN-γ (5 μg/ml), IL-2 (50 U/ml) and anti-IL-4 (anti-IL4 at 10% culture supernatant)	Butyrate (1 mM) Pentanoate (0.5-5 mM)	3-day treatment during polarization	Pentanoate hindered polarization and inhibited the production of IL-17A Pentanoate does not promote expansion of Tregs, but Butyrate does (CD4+FoxP3+ cells)	HDAC inhibition Pentanoate acting as acetyl-CoA precursor Increased mTOR function
Luu et al. (27)	CD8+ T cells CD8+ CAR-T cells	Sp and LN C57BL/6 SPF mice, hPBL	CTLs: p-c anti-CD3 (5 μg/ml), soluble anti-CD28 (1 μg/ml) Murine CAR-T: p-c anti-CD3 (2 μg/ml), soluble anti-CD28 (2 μg/ml) Human CAR-T: CD3/CD28 Dynabeads	CTLs: IL-2 (50 U/ml), anti-IFN-γ (10 μg/ml) Murine CAR-T: IL-2 (50 U/ml), IL-7 (10 ng/ml) and IL-15 (10 ng/ml) Human CAR-T: human IL-2 (100 U/ml) - Dynabead removal after 5 days	Acetate (2 mM) Propionate (2 mM) Butyrate (0.5 mM) Pentanoate (2 mM)	3-day treatment during polarization	Butyrate and Pentanoate enhance the production of effector molecules such as CD25, IFN-γ and TNF-α and enhance anti-tumor activity of CTLs and CAR T cells.	Increased mTOR function HDAC class I inhibition
He et al. (28)	CD8+ T cells	LN OT-I mice (OVA specific) hPBL	p-c anti-CD3 (1μg/ml), soluble anti-CD28 (2μg/ml)	IL-2 (10 ng/ml)	Propionate (5 mM) Butyrate (1 mM)	12 hours after 2 days of activation	Butyrate and Propionate promote IFN-γ and Granzyme B production in CD8+ T cells Butyrate and Propionate boosts antitumor cytotoxic effect	HDAC inhibition ID2-dependent IL-12 signaling

Sp, spleen; LN, Lymph node; hPBL, human peripheral blood lymphocytes; p-c, plate-coated; CTLs, cytotoxic T Lymphocytes; CAR, chimeric antigen receptor; IL, interleukin.

## Author contributions

Conceptualization and Original Draft Preparation, MU-H. Writing—Review and Editing AG-B and MU-H All authors contributed to the article and approved the submitted version.
